# Genetic Variants of the *FADS* Gene Cluster and *ELOVL* Gene Family, Colostrums LC-PUFA Levels, Breastfeeding, and Child Cognition

**DOI:** 10.1371/journal.pone.0017181

**Published:** 2011-02-23

**Authors:** Eva Morales, Mariona Bustamante, Juan Ramon Gonzalez, Monica Guxens, Maties Torrent, Michelle Mendez, Raquel Garcia-Esteban, Jordi Julvez, Joan Forns, Martine Vrijheid, Carolina Molto-Puigmarti, Carmen Lopez-Sabater, Xavier Estivill, Jordi Sunyer

**Affiliations:** 1 Center for Research in Environmental Epidemiology (CREAL), Barcelona, Catalonia, Spain; 2 Hospital del Mar Research Institute (IMIM), Barcelona, Catalonia, Spain; 3 CIBER Epidemiología y Salud Pública, Barcelona, Catalonia, Spain; 4 Genetic Causes of Disease Group, Genes and Disease Program, Center for Genomic Regulation (CRG), Barcelona, Catalonia, Spain; 5 Area de Salud de Menorca, IB-SALUT, Menorca, Spain; 6 Department of Nutrition and Food Science, Faculty of Pharmacy, University of Barcelona, Barcelona, Catalonia, Spain; 7 Genetics Unit, Department of Health and Experimental Life Sciences, Pompeu Fabra University (UPF), Barcelona, Catalonia, Spain; 8 Department of Experimental and Health Sciences, Pompeu Fabra University, Barcelona, Catalonia, Spain; Hospital Universitario 12 de Octubre, Spain

## Abstract

**Introduction:**

Breastfeeding effects on cognition are attributed to long-chain polyunsaturated fatty acids (LC-PUFAs), but controversy persists. Genetic variation in fatty acid desaturase (FADS) and elongase (ELOVL) enzymes has been overlooked when studying the effects of LC-PUFAs supply on cognition. We aimed to: 1) to determine whether maternal genetic variants in the *FADS* cluster and *ELOVL* genes contribute to differences in LC-PUFA levels in colostrum; 2) to analyze whether these maternal variants are related to child cognition; and 3) to assess whether children's variants modify breastfeeding effects on cognition.

**Methods:**

Data come from two population-based birth cohorts (n = 400 mother-child pairs from INMA-Sabadell; and n = 340 children from INMA-Menorca). LC-PUFAs were measured in 270 colostrum samples from INMA-Sabadell. Tag SNPs were genotyped both in mothers and children (13 in the *FADS* cluster, 6 in *ELOVL2*, and 7 in *ELOVL5*). Child cognition was assessed at 14 mo and 4 y using the Bayley Scales of Infant Development and the McCarthy Scales of Children's Abilities, respectively.

**Results:**

Children of mothers carrying genetic variants associated with lower FADS1 activity (regulating AA and EPA synthesis), higher FADS2 activity (regulating DHA synthesis), and with higher EPA/AA and DHA/AA ratios in colostrum showed a significant advantage in cognition at 14 mo (3.5 to 5.3 points). Not being breastfed conferred an 8- to 9-point disadvantage in cognition among children GG homozygote for rs174468 (low FADS1 activity) but not among those with the A allele. Moreover, not being breastfed resulted in a disadvantage in cognition (5 to 8 points) among children CC homozygote for rs2397142 (low ELOVL5 activity), but not among those carrying the G allele.

**Conclusion:**

Genetically determined maternal supplies of LC-PUFAs during pregnancy and lactation appear to be crucial for child cognition. Breastfeeding effects on cognition are modified by child genetic variation in fatty acid desaturase and elongase enzymes.

## Introduction

Brain development depends on the combined effects of incompletely understood genetic and environmental factors. The majority of dry weight in an adult brain is composed of lipids, 35% of which are long chain polyunsaturated fatty acids (LC-PUFAs) [1]. During development the brain is more highly enriched than most other tissues in LC-PUFAs that are essential for efficient neurogenesis, myelination, neurite outgrowth, dendritic arborisation and neurotransmission [2]–[7]. During pregnancy, the fetus is supplied with preformed maternal LC-PUFAs by placental transfer. After birth breast milk provides a unique supply of crucial LC-PUFAs including eicosapentaenoic acid (EPA), arachidonic acid (AA) and docosahexanoic acid (DHA), which support the accretion of LC-PUFA in the brain growth spurt that takes place during the third trimester of pregnancy and the first two years of life [8], [9]. Higher concentrations of DHA and AA, rapidly incorporated in the developing brain, are found in infants who are breastfed in comparison with infants fed with unsupplemented formulas [10]. However, it remains controversial whether breastfeeding provides a direct nutritional advantage for child cognition– attributed in part to the high levels and range of LC-PUFAs– or simply reflects more favorable socio-environmental factors– no fully controlled in statistical analysis– among women who breast feed [11]–[14].

LC-PUFA synthesis is controlled by key enzymes (desaturases and elongases) encoded by the *FADS* gene cluster and the *ELOVL* gene family (**see Supporting Information Figure S1**). Fatty acid desaturases are encoded by *FADS1* and *FADS2* genes, which build a gene cluster together with a third desaturase gene, *FADS3* [15]. *FADS2* is the rate-limiting step on the metabolic pathway leading to AA and DHA production [16]. Genes involved in the elongation of LC-PUFAs include *ELOVL4* (specifically expressed in human retina), and *ELOVL2* and *ELOVL5* that are expressed in brain [17]. Polymorphisms of the *FADS* gene cluster and *ELOVL2* gene have been reported to be associated with plasma variation in LC-PUFA levels [18]–[22]. In addition, in the absence of dietary supplies, desaturation and elongation processes may play a role in ensuring supply of LC-PUFAs such as DHA and AA [23].

Research on the effects of LC-PUFA supply during pregnancy and lactation taking into account maternal and child inter-individual genetic variation in fatty acid desaturase and elongase activities may be critical to elucidate whether breastfeeding influences cognitive development as a unique source of LC-PUFAs, independent of other risk factors [24]. Unlike breastfeeding practises, genotype is not likely to be influenced by social, behavioural or lifestyle variables. Therefore, using the principles of Mendelian randomization, associations between the functional polymorphism of *FADS* and *ELOVL* genes and child cognition are unlikely to be confounded by social or behavioral factors and are more likely to be representative of a causal relationship. The aims of this study were: 1) to determine whether maternal genetic variants in the *FADS* cluster and *ELOVL* genes contribute to differences in LC-PUFA levels in first breast milk (colostrum); 2) to analyze whether these maternal variants are related to cognitive outcomes in the child; and 3) to assess whether children's variants in these genes modify breastfeeding effects on cognition. To achieve these objectives we used data from two Spanish population-based birth cohorts included in the INMA (INfancia y Medio Ambiente [Environment and Childhood]) Project.

## Methods

### Ethics statement

Written informed consent was obtained from all participants and the study was approved by the Clinical Research Ethical Committee of the Municipal Institute of Health Care (CEIC-IMAS), Barcelona.

**Figure 1:**
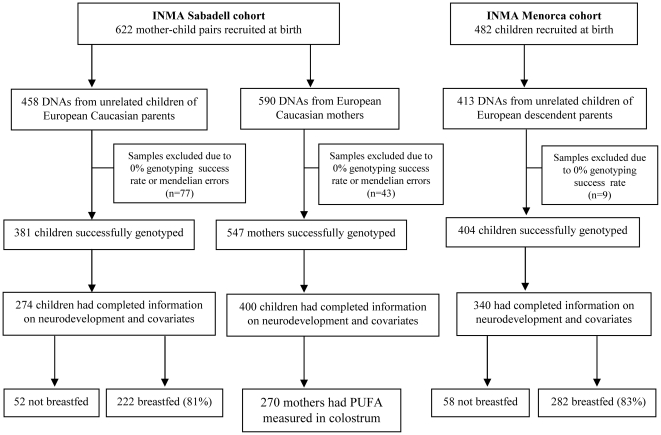
Flow diagram for study design and participant selection.

Study design and participants are summarized in Figure 1. Distribution of child and maternal characteristics of participants and non participants are showed in the Online Repository (**see Supporting Information Table S5**).

### The INMA-Sabadell birth cohort

The INMA-Sabadell cohort was established in Sabadell (Barcelona, Spain) between 2004 and 2006 [25]. A total of 657 women were enrolled at the first trimester of pregnancy (participation rate was 60%, 657 of 1097 eligible women), and 622 (94%) followed until the child's birth. Detailed information about child feeding was completed by interviewer-administered questionnaires with mothers at 6 and 14 months. Exclusively breastfeeding was defined as receiving breast milk only but allowing supplementation of non-milk liquids according to the WHO's definition [26]. Mental development was assessed at age 14 months (range 12–17 months) by 2 specially trained psychologists using the Bayley Scales of Infant Development, first edition [27], [28]. The Bayley Scale for Cognitive Development is composed by two scales, the mental development and psychomotor. For the present study, the main outcome was the score in the mental development scale consisting in 163 items that assess age-appropriate cognitive development in areas such as performance ability, memory, and first verbal learning.

### The AMICS INMA-Menorca birth cohort

The Menorca Asthma Multicenter Infant cohort Study (AMICS) is a population-based birth cohort established in the island of Menorca (Spain) in 1997–98. A total of 482 children were recruited at birth from 492 pregnant women residing on the island [29]. Breastfeeding was assessed by interviewer-administered questionnaires to mothers three times at ages 6 months, 14 months, and 2 years[30]. Infants not breastfeed received formula feeding at a time before LC-PUFA supplementation of formula became widely available in Spain. Two certified psychologists performed the neuropsychological testing of the children at age 4 years measuring cognitive functioning with the Spanish version of the McCarthy Scales of Children's Abilities (MCSA), consisting of 5 sub-scales assessing several cognitive domains (verbal, perceptual-performance, quantitative, memory, and motor) and a general cognitive index [31]–[33]. For the present study score in the general cognitive index was used as the main outcome.

### Genotyping procedure

All genotyping processes were performed at the Barcelona Node of the Spanish National Genotyping Centre (CEGEN). In the INMA-Sabadell cohort maternal DNA samples were extracted from blood obtained at first trimester of pregnancy. Child DNA samples were extracted from cord blood obtained at birth in the INMA-Sabadell cohort (100%) and from blood (85%) obtained at age 4 in the INMA-Menorca cohort (15 % from saliva). Details of the SNP selection process are provided in the supplementary information material (**see Supporting information Appendix S1**). Rs numbers, gene location, minor allele frequencies and Hardy-Weinberg Equilibrium values are shown in the Online Repository (**see Supporting Information Table S1**). Genotyping was performed blind to measures of breastfeeding and LC-PUFA exposure, as well as, cognition scores. Finally, twenty-six SNPs out of 36 initially selected were successful genotyped and included in the analysis (13 in the *FADS* gene cluster, 6 in *ELOVL2* gene, and 7 in *ELOVL5* gene). Linkage disequilibrium (LD) parameters (D' and r2) were estimated using the Haploview software (**see Supporting Information Table S6, Table S7, Table S8**).

### Measurement of colostrum LC-PUFA

Colostrum samples were obtained from the first 270 willing participants from the INMA-Sabadell cohort. Samples were collected during the first 48–96 hours post-partum, transported to the laboratory in ice boxes less than 2 hours after collection and stored at −80°C until analyzed. The following fatty acids were identified and quantified according to the method developed by Molto-Puigmarti et al. [34]: linoleic acid (LA), α-linolenic acid (ALA); AA; eicosatrienoic acid (DGLA); EPA; docosapentaenoic acid (DPA) and DHA. The ratio between the concentrations of the product-substrate pairs of the desaturase and elongase reactions have been previously used as strong indicators for the efficiency of the enzymes and as a proxy for enzymatic activity [35]. Thus, we further calculated the following ratios as surrogate indexes of FADS and ELOVL5 enzymatic activities: AA:DGLA for FADS1, DGLA:LA and DHA:DPA for FADS2 as previously described [36], [37], and DPA:EPA for ELOVL5 activity. In addition, ratios EPA/AA and DHA/AA, which reflect the proportions of key n-3 to n-6 fatty acids essential for brain growth and function, were calculated.

### Statistical analysis

The outcomes of interest, general cognitive index from McCarthy test and the mental development scale score from the Bayley test, were standardized to a mean of 100 corresponding to the mean of the raw scores, and standard deviation of 15. LC-PUFAs were log-transformed to achieve a normal distribution. Bivariate analyses exploring how breastfeeding behaviors and colustrum LC-PUFA profiles were related to either child cognition or to genetic variants used χ^2^-tests for categorical data and Krukall-Wallis tests for continuous data. For genetic variables, departure from HWE within mothers and children was evaluated using a χ^2^-test.

Associations between maternal genetic variants and both child cognition and LC-PUFA levels and ratios in colostrum were assessed using multivariable linear models and evaluated using likelihood ratio tests. The resultant regression coefficients represent the mean difference in cognitive scores or LC-PUFA levels between genotype groups. All variables significantly associated with general cognitive scores in the bivariate models (p<0.2) were included in the multivariate model, and only retained if they had an at least marginally significant association (p<0.1) or modified the coefficient by at least 5% [32], [33], [38]. Final multivariate models were adjusted for sex, child age (days), psychologist, quality of neuropsychological test, maternal education, breastfeeding, and use of gas stove at home. For associations between LC-PUFA and maternal genotype, unadjusted results are reported since incorporating potential confounders in multivariable linear models including maternal age, body mass index, smoking habits, and maternal diet intake of fish, seafood, meat and egg intakes measured by a 101-item validated food frequency questionnaire during pregnancy did not meet criteria for confounding (change-in-estimate ≥5%), and finally were excluded for parsimony. Bonferroni correction was applied to take multiple testing into account and p value thresholds were set at 0.002 (0.05/23).

We tested interactions between child's genetic variants and breastfeeding, as well as, between LC-PUFA levels and n-3/n-6 ratios in colostrum. Linear models were also used to assess whether children's genetic variants modified the effects of breastfeeding behaviors on cognitive test scores, adjusting for other covariables. Similarly, linear models were used to assess whether children's genetic variants modified the effects of colostrum LC-PUFA profiles on cognitive scores. For these analyses, selected single LC-PUFA and n-3/n-6 ratios were dichotomized as close as possible to p = 0.50, and analyses were restricted to breastfed children. The resultant regression coefficient gives the mean difference in cognitive scores between the two groups (e.g. children exposed to low vs. high colostrum fatty acid concentrations or children with contrasting genetic variants), having controlled for confounders. Statistical analysis was performed with R statistical package version 2.9.1 using the SNPassoc package [39].

## Results

### Associations between maternal *FADS* gene cluster and *ELOVL* gene family SNPs and LC-PUFA profiles in colostrum

Associations between maternal genetic variants and LC-PUFA levels in colostrum are showed in the Online Repository (**see Supporting Information Table S2**). The rs174537-T, rs174570-T, rs2072114-G, rs174602-G, rs526126-G, rs174626-C, rs174464-T, and rs174468-G alleles in the *FADS* cluster were related to lower levels of AA (Table S2). The rs174602-G, and 174464-T in the *FADS* cluster were associated with lower levels of DHA, and other SNPs in the cluster showed a trend to be associated with DHA levels in the same direction as the observed for AA (Table S2). Regarding elongases rs953413-A and rs3798719-T in *ELOVL2* gene were associated with higher EPA levels. None of the SNPs in the *ELOVL5* were associated with AA, EPA or DHA levels at a p value of 0.05, although a trend was observed for rs12207094 and EPA levels. Finally, several SNPs in the *FADS* cluster (rs174464 and rs174468), *ELVOL2* (rs3734397, rs953413 and rs3798719) and *ELVOL5* (rs17544159, rs9395855 and rs12207094) genes were associated with EPA/AA ratio and/or DHA/AA ratio (Table S2).

Table 1 shows the associations observed between the *FADS* cluster and *ELOVL* gene family SNPs and enzymatic indexes estimated based on LC-PUFA profiles. The rs174537-T, rs968567-A, rs2072114-G, rs526126-G, rs174626-C, rs174627-T, rs174464-T, rs174468-G alleles were associated with lower FADS1 index. Three of the previous SNPs (rs174537, rs968567 and rs174627) were inversely associated with FADS2 index. Moreover, the rs174602-G allele was associated with lower FADS2 index (capacity to synthesize DHA from EPA). In *ELOVL5*, the rs2397142-C allele was nominally associated with lower capacity to metabolize EPA to DPA.

**Table 1:** FADS1, FADS2 and ELOVL5 enzymatic indexes according to maternal genotypes and LC-PUFA levels in colostrum.

			11		12		22		
		Major/minor allele	N	Mean (sd)	N	Mean (sd)	N	Mean (sd)	p value^*^
**FADS1 index**									
**AA:DGLA**									
Gene	SNP								
* FADS*	rs174537	G/T	142	1.46 (0.02)	109	1.22 (0.03)	19	0.96 (0.08)	1.0×10^−20^
* FADS*	rs968567	G/A	200	1.44 (0.02)	57	1.04 (0.03)	3	0.76 (0.08)	2.4×10^−29^
* FADS*	rs2072114	A/G	219	1.35 (0.02)	49	1.23 (0.04)	2	0.93 (0.04)	0.0026
* FADS*	rs526126	C/G	160	1.37 (0.02)	66	1.25 (0.04)	2	1.06 (0.19)	0.0031
* FADS*	rs174626	T/C	66	1.43 (0.03)	141	1.32 (0.02)	63	1.25 (0.04)	0.0007
* FADS*	rs174627	C/T	209	1.37 (0.02)	56	1.20 (0.04)	5	0.99 (0.14)	7.5×10^−06^
* FADS*	rs174464	C/T	116	1.41 (0.03)	103	1.26 (0.03)	19	1.20 (0.07)	7.2×10^−05^
* FADS*	rs174468	G/A	100	1.28 (0.03)	124	1.34 (0.03)	40	1.43 (0.04)	0.0078
**FADS2 indexes**									
**DGLA:LA**									
Gene	SNP								
* FADS*	rs174537	G/T	142	0.06 (0.001)	109	0.07 (0.002)	19	0.09 (0.008)	8.5×10^−06^
* FADS*	rs968567	G/A	200	0.06 (0.001)	57	0.09 (0.003)	3	0.11 (0.016)	1.1×10^−15^
* FADS*	rs174627	C/T	209	0.06 (0.001)	56	0.07 (0.003)	5	0.08 (0.020)	0.0129
**DHA:EPA**									
Gene	SNP								
* FADS*	rs174602	A/G	209	1.65 (0.03)	56	1.56 (0.03)	5	1.42 (0.05)	0.0039
**ELOVL5 index**									
**DPA:EPA**									
Gene	SNP								
* ELOVL5*	rs2397142	C/G	125	7.81 (0.31)	112	8.08 (0.42)	31	10.02 (1.28)	0.0362

INMA Sabadell cohort.

Only statistically significant associations are showed.

Major allele:1; minor allele:2. Data are means (standard error). *P-values for additive genetic models assuming a trend per copy of the minor allele.

AA: Arachidonic acid; DGLA: Eicosatrienoic acid; LA: Linoleic acid; DHA: Docosahexaenoic acid; DPA: Docosapentaenoic acid; EPA: Eicosapentanoic acid.

### Associations between maternal polymorphisms in the *FADS* gene cluster and *ELOVL* genes and child cognition

In the *FADS* gene cluster, we found that maternal SNPs rs968567, rs174602, rs174627 and rs174464 were nominally associated with child cognition (Table 2). In general the allele associated with higher child cognition scores were the alleles associated with high FADS2 index (the limiting enzyme of the pathway), which correspond to low FADS1 index (as both enzymatic ratios are estimated using DGLA levels). The most significant association found was for the maternal SNP rs174627 (p<0.001). In particular children of mothers carrying at least one copy of the minor allele (T) for this variant had higher cognition scores compared to children of mothers homozygotes for the major allele (Table 2). None of the SNPs in the *ELOVL2* gene showed a significant association with child cognition. Regarding the *ELOVL5* gene, we found that children of mothers carrying the rs17544159-C allele (associated with higher EPA/AA ratio in colostrums) or carrying the rs12207094-T allele (associated with both higher EPA/AA and DHA/AA ratios in colostrum) had higher cognition scores related to children of mothers homozygote for the major allele.

**Table 2:** Association between maternal genetic variants and child cognition at age 14 months.

			11		12		22		
	N	Major/minor allele	N	Score	N	Score	N	Score	p value*
***FADS*** ** cluster**									
rs174537	400	G/T	203	99.5 (1.1)	169	101.0 (1.2)	28	100.7 (2.5)	0.130
rs968567	385	G/A	296	99.4 (0.9)	83	102.6 (1.6)	6	106.3 (4.7)	0.023
rs174570	347	C/T	279	101.0 (0.9)	64	98.5 (2.0)	4	95.8 (3.8)	0.330
rs2072114	399	A/G	310	100.2 (0.9)	87	100.5 (1.5)	2	100.1 (5.7)	0.880
rs174602	393	A/G	230	101.3 (0.9)	137	98.8 (1.4)	26	98.3 (2.9)	0.027
rs526126	336	C/G	234	99.9 (1.0)	99	102.8 (1.6)	3	105.8 (8.3)	0.059
rs174626	399	T/C	102	99.9 (1.4)	209	99.5 (1.1)	88	102.2 (1.7)	0.240
rs174627	399	C/T	313	99.4 (0.9)	82	103.1 (1.5)	4	109.4 (10.3)	<0.001
rs7482316	382	A/G	310	100.7 (0.9)	70	98.0 (2.0)	2	97.2 (20.1)	0.474
rs174464	348	C/T	174	98.3 (1.2)	149	102.2 (1.2)	25	106.6 (2.2)	0.008
rs174468	392	G/A	141	100.4 (1.4)	187	100.1 (1.1)	64	99.9 (1.8)	0.563
***ELOVL2***									
rs3734397	400	A/G	212	100.9 (0.9)	168	99.1 (1.2)	20	102.7 (4.5)	0.291
rs953413	387	G/A	114	99.2 (1.4)	193	101.1 (1.1)	80	100.0 (1.7)	0.398
rs10498676	392	G/A	288	100.0 (0.9)	94	101.7 (1.6)	10	92.6 (7.6)	0.583
rs6936315	338	T/C	241	100.5 (1.0)	89	101.0 (1.7)	8	102.2 (4.1)	0.844
rs3798719	385	C/T	196	99.7 (1.2)	151	101.6 (1.2)	38	96.3 (2.2)	0.849
rs13204015	389	T/C	356	100.7 (0.8)	32	98.1 (3.1)	1	97.2 (0.0)	0.309
***ELOVL5***									
rs17544159	383	A/C	332	99.4 (0.8)	51	105.1 (2.0)	-	-	0.016
rs2281274	394	T/C	210	99.7 (1.1)	147	100.6 (1.3)	37	101.7 (2.4)	0.275
rs2294859	392	T/C	332	100.8 (0.9)	56	99.2 (1.7)	4	92.9 (8.3)	0.983
rs9395855	399	T/G	105	98.3 (1.6)	204	101.0 (1.0)	90	100.8 (1.7)	0.633
rs11968589	390	C/T	310	100.5 (0.9)	74	99.1 (1.9)	6	96.2 (5.6)	0.601
rs2397142	397	C/G	173	99.7 (1.1)	178	100.1 (1.2)	46	101.9 (2.1)	0.499
rs12207094	398	A/T	287	99.1 (0.9)	106	102.8 (1.4)	5	119.1 (7.4)	0.003

INMA Sabadell cohort.

Major allele:1; minor allele:2. Data are means (standard error, SE).

*P value for the additive genetic models assuming a trend per copy of the minor allele.

All models are adjusted for sex, child age (days), psychologist, quality of neuropsychological test, maternal education, breastfeeding, and use of gas stove at home.

### Associations between child polymorphisms in the *FADS* gene cluster and *ELOVL* genes and child cognition

Regarding child genetic variants, after Bonferroni correction no statistically significant associations were found between child polymorphisms and cognition, and we could no replicate the nominal associations found in Menorca cohort (**see Supporting Information Table S3**).

### Child polymorphisms in the *FADS* cluster and *ELOVL5* gene modify breastfeeding effect on cognition

**Figure 2:**
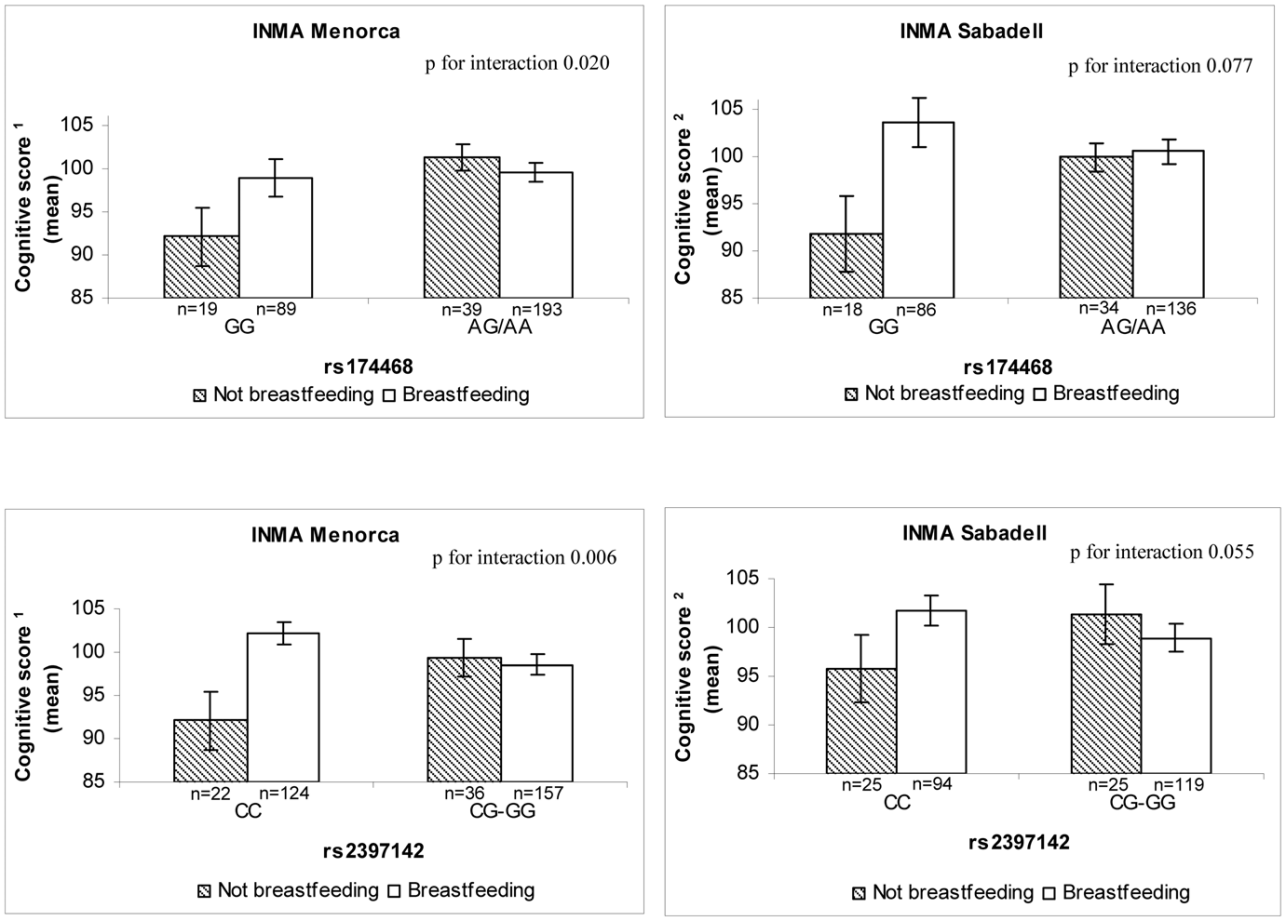
Child cognition scores by child' genetic SNPs in the *FADS* gene cluster (rs174468) and *ELOVL5* gene (rs2397142), by breastfeeding and cohort. Bars represent standard error. ^1^Adjusted for sex, school trimester at testing, maternal social class, maternal education, maternal smoking and alcohol consumption in pregnancy, indoor nitrogen dioxide (NO2) levels. ^2^Adjusted for sex, child age (in days), psychologist, quality of neuropsychological test, maternal education, and use of gas stove at home.

We found two statistically significant gene-breastfeeding interactions that were replicated in both cohorts. We found that not being breastfed conferred a disadvantage in cognition (9 point in the INMA-Menorca cohort and 8 points in the INMA-Sabadell cohort) among children GG homozygotes for rs174468 (low FADS1 index), but not among those carrying at least one copy of the A allele (high FADS1 index) (Figure 2). In addition, not being breastfed resulted in a disadvantage in cognition (8-point in INMA-Menorca and 5-point in the INMA-Sabadell) among children CC homozygotes for rs2397142 (low ELOVL5 index), but not among those carrying at least one copy of the G allele (high ELOVL5 index). In contrast, breastfed children did not differ in cognition score irrespective of their genetic variants in these polymorphisms.

Children' genotypes were not related to potential confounders including maternal social class, maternal education level, reproductive outcomes, diet intakes, or maternal tobacco and alcohol consumption during pregnancy. In addition, the prevalence of breastfeeding was similar across children with different genotypes (**see Supporting Information Table S4**).

### Child polymorphism in *ELOVL5* gene modifies the effect of colostrum LC-PUFA levels on cognition

In the INMA-Sabadell cohort we analyzed whether child genetic variants interacted with LC-PUFA levels in colostrum to influence cognition. Among breastfed children carrying at least one G allele for rs2397142 (high ELOVL5 index), there was no association between cognition and LC-PUFA levels in colostrum. In contrast, among children CC homozygotes for rs2397142 (low ELOVL5 index), exposure to high levels of EPA, and high EPA/AA and DHA/AA ratios were associated with a 6.7-, 8.6- and 10.1-points advantage in scores, respectively, relative to children exposed to low levels (Table 3).

**Table 3:** Child cognition scores* (mean and standard error (SE)) by levels of LC-PUFA in colostrum and by rs2397142 (*ELOVL5* gene) among breast fed children of the INMA Sabadell cohort.

	rs2397142 CC			rs2397142 CG-GG			
	N	Mean	SE	N	Mean	SE	p for interaction
**EPA/AA ratio**							
Low	25	96.1	3.5	41	100.7	2.3	0.029
High	40	104.8	2.4	37	98.0	2.9	
**DHA/AA ratio**							
Low	35	96.8	2.8	39	98.6	2.7	0.018
High	30	106.9	2.7	39	100.2	2.4	

AA: Arachidonic acid; EPA: Eicosapentanoic acid; DHA: Docosahexaenoic acid.

* Adjusted for sex, age (days), psychologist, quality of neuropsychological test, maternal education, and use of gas stove at home.

## Discussion

In the present study, we analysed polymorphisms in genes encoding the key enzymes involved in LC-PUFA synthesis in mothers and their offspring to disentangle their role in modifying potential nutritional advantages of breastfeeding on child cognition. Maternal genetic variants in the *FADS* gene cluster and *ELOVL5* gene were associated with higher colostrum levels of n-3 LC-PUFA (i.e. EPA and DHA) as well as with higher cognitive scores in their children. In infants, we found that genetic variants in the *FADS* cluster and *ELOVL5* gene modified the effects of breastfeeding on cognition. Children with variants associated with lower synthesis of LC-PUFA had higher scores when breastfed, while those with greater capacity to synthesize these fatty acids had higher scores regardless of breastfeeding practises. We confirmed these gene-breastfeeding interactions in two independent population-based birth cohorts. Moreover, we ruled out the possibility that these findings were due to potential confounders including correlations between genes and exposures, intrauterine growth differences, dietary or sociodemographic factors.

### Maternal genetic variation in LC-PUFA metabolizing enzymes, colostrum LC-PUFA profile and child cognition

Although fatty acid desaturase activities are present in the mammary gland [40], few studies have assessed whether maternal polymorphisms in key enzymes involved in LC-PUFA synthesis influence the composition of breast milk [20], [22], and the effects on child phenotypes such as cognition have not been investigated so far. To this purpose we used LC-PUFA measurements from early breast milk (colostrum), since its LC-PUFA composition is related to the LC-PUFA composition of brain tissue in the newborn [41]. Our results on the direction of the association between maternal genetic variants in desaturases and LC-PUFA levels in colostrum are consistent with previous findings showing associations between several SNPs in the *FADS* cluster and plasma, erythrocyte and breast milk LC-PUFA levels in adults [18]–[20], [22], [40]. In our study, the two maternal *FADS* cluster SNPs most strongly related to disparities in colostrum LC-PUFA levels were rs174537 previously reported to be associated with LC-PUFA levels in genome-wide association studies [18], and rs968567 recently reported to influence *FADS2* transcription in an *in vitro* system [42]. We also identified other *FADS* cluster SNPs significantly related to variation in colostrum LC-PUFA levels as well as with FADS enzymatic indexes previously described [20], [37]. In general, minor alleles in the *FADS* cluster (associated with low FADS1 index and high FADS2 indexes) were related to higher child cognitive scores, although no statistically significant associations were found for all of them. FADS2 is considered the limiting enzyme which strengthens the importance of the present results. Since not all the LC-PUFAs were measured, we could not evaluate the same ratios in the n-3 pathway. However, the fact that both types of LC-PUFA compete for the same enzymes system, suggests that similar results are expected in the n-3 pathway. In addition, although tissue LC-PUFA levels are also determined by dietary intake, associations between the analyzed SNPs and both LC-PUFA colostrum levels and desaturase and elongase enzymatic activity estimates did not change after taking into account information on maternal diet habits including fish, seafood, meat and eggs intakes measured by food frequency questionnaire during pregnancy.

AA and DHA have been proposed to play important roles in synaptic transmission and plasticity during early brain development [8]. Furthermore, the imbalance between n-6 and n-3 fatty acids has been suggested to be related to neuropsychiatric disorders in children [8]. Although, infants are capable of synthesizing DHA and AA accumulation of LC-PUFA fatty acids *in utero* are derived predominantly through placental transfer [43]. Based on the present results, maternal genetic variants influencing fatty acid desaturation and elongation activities– and thus levels of LC-PUFA in colostrum– appear to have functional importance for infant brain development. In this regard, we found that children of mothers with genetically determined lower levels of AA, higher EPA/AA, and higher capacity to synthesize DHA and therefore higher DHA/AA ratios, showed a significant advantage in cognition at age 14 months.

### Child polymorphisms in LC-PUFA metabolizing enzymes, breastfeeding and child cognition

After birth, formula fed infants depend on the utilization of body stores or on the endogenous ability to synthesize LC-PUFAs: the latter may help that may compensate for deficient supplies of LC-PUFAs associated with not being breastfed. We found that genetic variants in the *FADS* gene cluster and *ELOVL5* gene in children modified the effect of breastfeeding on cognition; and results were replicated in both cohorts. Not being breastfed conferred a disadvantage in cognition among children with lower FADS1 index, and similarly, not being breastfed conferred a disadvantage among children with lower ELOVL5 index. These results of the *FADS* cluster variant are in general accordance with those reported by Caspi et al. [44]. As proposed by these authors the genetic modification of breastfeeding effects on cognition is unlikely to be directly caused by the analyzed SNPs, but rather mediated by more efficient LC-PUFA processing.

Biological effects of LC-PUFAs on brain function are assumed to be mediated by the availability of LC-PUFA with >20 carbon atoms and >3 double bonds (i.e. AA, EPA and DHA) [8]. In mammals, *ELOVL5* catalyzes the two carbon elongation process required for the synthesis of DHA from EPA [45]. The modification of breastfeeding effects on child cognition by a SNP in *ELOVL5* found in the present study could be explained by higher capacity in infants with this variant to synthesize 20 carbon fatty acids from their precursors provided by breast milk. While previous studies have focused primarily on the *FADS* gene cluster, interestingly we found that breastfed children who were CC homozygotes (low ELOVL5 index) exposed to higher EPA, EPA/AA and DHA/AA in colostrum had a significant advantage in cognition in comparison to those exposed to lower concentrations. These results suggest that homozygosity for the C allele could be related to less efficient synthesis of DHA from EPA, probably due to decreased transcription or to a less enzyme activity. On the contrary, the presence of at least one G allele could be related to more efficient fatty acid metabolism, which could compensate for deficient supplies of LC-PUFAs derived from not being breastfed or being exposed to breast milk containing lower levels of EPA and DHA. Consistent with this mechanism, experimental studies in baboon neonates have revealed that exposure to different levels of DHA in formulas induced global changes in gene expression in cerebral cortex [46], and only a single elongation enzyme was differentially expressed: the analogue to human ELOVL5.

### Implications for genetic neuroscience and newborn nutrition

Identifying genes for variation in the range of normal cognitive functioning could help in understanding biological mechanisms and pathways of milder but more-prevalent forms of impaired neurofunctioning, which are often associated with autism, schizophrenia, and attention deficit hyperactivity disorder (ADHD). Complex traits, such as cognition, are the result of combined effects of genetic and environmental factors[47]; however, gene-environment interactions are repeatedly overlooked in genome-wide scans which could result in unexplained heritability [48]. In addition, the results indicate that breastfeeding offers a real nutritional advantage for child cognition, and that this advantage is related to LC-PUFA profiles. The ratios between n-3 and n-6 fatty acids, particularly EPA/AA and DHA/AA, have been emphasized in infant nutrition [8]. Significant improvement in ADHD symptoms have been observed with a significant increase in EPA and DHA supplies, as well as a drop in the AA/EPA ratio [49]. Also a significant association between a SNP in the *FADS2* gene and ADHD has been reported (carriers of the genetic variant associated with ADHD have greatly elevated AA contents in their serum phosplipids) [50]. These earlier studies and findings from the present study suggest not only a need to increase n-3 (EPA and DHA) in the diet, including in infant formulae, but also perhaps to decrease the n-6 content (i.e. AA). Furthermore, failure to consider genetic heterogeneity in LC-PUFA metabolism in participants may dilute supplementation effects and as a result inconsistent conclusions are derived from randomized controlled trials.

### Strengths and limitations

Strengths of this study include the use of data from two independent population-based birth cohorts. We also had both maternal and child genetic information. Cognitive assessment was measured with validated instruments. The observed associations are unlikely to be due to population stratification since the discovery and replication samples were restricted to Caucasian individuals of European origin recruited in Catalonia and in Menorca. Furthermore, the frequency distribution of minor and major alleles was similar in both cohorts and to that listed in the HapMap CEU sample. In comparison with the study by Caspi et al., we assessed the association of both maternal and child genetic variation in fatty acid desaturases and elongases on cognition. Moreover we assessed the genetic variability in the whole *FADS* cluster gene and also in elongase genes involved in the LC-PUFA synthesis pathway. In addition, the use of biomarkers such as colostrum levels of LC-PUFA strengthens the findings.

Among the study limitations we acknowledge the small sample size that has probably limited power to detect associations with small effect sizes and associations with rare variants. Since we measured cognitive function at two different ages, phenotypic heterogeneity between cohorts may have limited our ability to detect some direct associations. However, our main aim was to seek for gene-environment interactions, which probably have a more relevant role in complex traits as cognition. In fact we were able to detect and replicate two gene-breastfeeding interactions with a plausible biological justification. Additionally, a proportion of mothers and children in both cohorts were not included in the present study because of non-participation in the neurodevelopment exam or failure in the genotyping. However, the analysis comparing participants and non-participants did not show any difference in general cognition or sociodemographic characteristics (**see Supporting Information Table S5**). We could no collect maternal DNA in Menorca cohort, thus associations between maternal genetic variants and child cognition could not be replicated and future studies are warranted.

### Conclusions

Results of the present study showed that LC-PUFA supplies during pregnancy and lactation, genetically determined by maternal desaturase and elongase activities, appear to have functional importance to the infant brain development. In addition, breastfeeding effects on cognition are also modified by child genetic variants in desaturase and elongase enzymes involved in the control of LC-PUFA pathways. Future research assessing the effects of breastfeeding and LC-PUFA diet supplementation on brain development should take into account genetic heterogeneity in key enzymes of LC-PUFA synthesis.

## Supporting Information

Appendix S1 Supporting methods.
(DOC)

Figure S1 The n-6 and n-3 fatty acid metabolism pathways.
(DOC)

Table S1 Characteristics of the SNPs analyzed in the *FADS* gene cluster, *ELOVL2* and *ELOVL5* genes. Abbreviations: SNP: single-nucleotide polymorphism; HWE: Hardy-Weinberg equilibrium; MAF: Minor allele frequency; ME: Mendelian errors.
(DOC)

Table S2 Associations* between maternal genetic variants in the *FADS* cluster, *ELOLV2* and *ELOVL5* genes and levels of LC-PUFA in colostrum. *p-value of the association using additive genetic models. Change in concentrations in the additive genetic models assuming a trend per copy of the minor allele. MAF: Minor allele frequency; AA: Arachidonic acid; EPA: Eicosapentanoic acid; DHA: Docosahexaenoic acid.
(DOC)

Table S3 Associations* between child polymorphisms in the *FADS* cluster, *ELOVL2* and *ELOVL5* genes and child cognition by cohort. *p values in the additive genetic models assuming a trend per copy of the minor allele. MAF: Minor allele frequency.
(DOC)

Table S4 Association of child genetic polymorphisms in the *FADS* cluster (rs174468) and *ELOVL5* (rs2397142) with general cognitive function score and selected covariates by cohort. ^¶^ Unless otherwise specified, p value derived from chi-2 test; §p value derived from Kruskall-Wallis test.
(DOC)

Table S5 Comparison of the distribution of child and maternal characteristics between children included and not included in the study by cohort. ^¶^ Unless otherwise specified, p value derived from chi-2 test; §p value derived from Kruskall-Wallis test.
(DOC)

Table S6 Pair-wise LD parameters (D'r2) in the *FADS* gene cluster in the INMA Menorca cohort (only SNPs included in the final analysis).
(DOC)

Table S7 Pair-wise LD parameters (D'/r2) in the *ELOVL2* gene in the INMA Menorca cohort (only SNPs included in the final analysis).
(DOC)

Table S8 Pair-wise LD parameters (D'/r2) in the *ELVOL5* gene in the INMA Menorca cohort (only SNPs included in the final analysis).
(DOC)

## References

[1] Haag M (2003). Essential fatty acids and the brain.. Can J Psychiatry.

[2] Uauy R, Hoffman DR, Peirano P, Birch DG, Birch EE (2001). Essential fatty acids in visual and brain development.. Lipids.

[3] Innis SM (2000). The role of dietary n-6 and n-3 fatty acids in the developing brain.. Dev Neurosci.

[4] He C, Qu X, Cui L, Wang J, Kang JX (2009). Improved spatial learning performance of fat-1 mice is associated with enhanced neurogenesis and neuritogenesis by docosahexaenoic acid.. Proc Natl Acad Sci U S A.

[5] Robson LG, Dyall S, Sidloff D, Michael-Titus AT (2010). Omega-3 polyunsaturated fatty acids increase the neurite outgrowth of rat sensory neurones throughout development and in aged animals.. Neurobiol Aging.

[6] Salvati S, Natali F, Attorri L, Di Benedetto R, Leonardi F (2008). Eicosapentaenoic acid stimulates the expression of myelin proteins in rat brain.. J Neurosci Res.

[7] Marza E, Lesa GM (2006). Polyunsaturated fatty acids and neurotransmission in Caenorhabditis elegans.. Biochem Soc Trans.

[8] Schuchardt JP, Huss M, Stauss-Grabo M, Hahn A (2010). Significance of long-chain polyunsaturated fatty acids (PUFAs) for the development and behaviour of children.. Eur J Pediatr.

[9] Sauerwald TU, Demmelmair H, Koletzko B (2001). Polyunsaturated fatty acid supply with human milk.. Lipids.

[10] Makrides M, Neumann MA, Byard RW, Simmer K, Gibson RA (1994). Fatty acid composition of brain, retina, and erythrocytes in breast- and formula-fed infants.. Am J Clin Nutr.

[11] Anderson JW, Johnstone BM, Remley DT (1999). Breast-feeding and cognitive development: a meta-analysis.. Am J Clin Nutr.

[12] Mortensen EL, Michaelsen KF, Sanders SA, Reinisch JM (2002). The association between duration of breastfeeding and adult intelligence.. JAMA.

[13] Der G, Batty GD, Deary IJ (2006). Effect of breast feeding on intelligence in children: prospective study, sibling pairs analysis, and meta-analysis.. BMJ.

[14] Simmer K, Patole SK, Rao SC (2008). Longchain polyunsaturated fatty acid supplementation in infants born at term.. Cochrane Database Syst Rev.

[15] Marquardt A, Stöhr H, White K, Weber BH (2000). cDNA cloning, genomic structure, and chromosomal localization of three members of the human fatty acid desaturase family.. Genomics.

[16] Sprecher H (2000). Metabolism of highly unsaturated n-3 and n-6 fatty acids.. Biochimica Et Biophysica Acta-Molecular and Cell Biology of Lipids.

[17] Jakobsson A, Westerberg R, Jacobsson A (2006). Fatty acid elongases in mammals: their regulation and roles in metabolism.. Prog Lipid Res.

[18] Tanaka T, Shen J, Abecasis GR, Kisialiou A, Ordovas JM (2009). Genome-wide association study of plasma polyunsaturated fatty acids in the InCHIANTI Study.. PLoS Genet.

[19] Schaeffer L, Gohlke H, Müller M, Heid IM, Palmer LJ (2006). Common genetic variants of the FADS1 FADS2 gene cluster and their reconstructed haplotypes are associated with the fatty acid composition in phospholipids.. Hum Mol Genet.

[20] Xie L, Innis SM (2008). Genetic variants of the FADS1 FADS2 gene cluster are associated with altered (n-6) and (n-3) essential fatty acids in plasma and erythrocyte phospholipids in women during pregnancy and in breast milk during lactation.. J Nutr.

[21] Martinelli N, Girelli D, Malerba G, Guarini P, Illig T (2008). FADS genotypes and desaturase activity estimated by the ratio of arachidonic acid to linoleic acid are associated with inflammation and coronary artery disease.. Am J Clin Nutr.

[22] Moltó-Puigmartí C, Plat J, Mensink RP, Müller A, Jansen E (2010). FADS1 FADS2 gene variants modify the association between fish intake and the docosahexaenoic acid proportions in human milk.. Am J Clin Nutr.

[23] Heird WC, Lapillonne A (2005). The role of essential fatty acids in development.. Annu Rev Nutr.

[24] Smith GD, Ebrahim S (2004). Mendelian randomization: prospects, potentials, and limitations.. Int J Epidemiol.

[25] Ribas-Fitó N, Ramón R, Ballester F, Grimalt J, Marco A (2006). Child health and the environment: the INMA Spanish Study.. Paediatr Perinat Epidemiol.

[26] World Health Organization (2008). Indicators for assessing infant and young child feeding practices: Part 1 Definitions..

[27] Bayley N (1977). Escalas Bayley de Desarrollo Infantil..

[28] Vrijheid M, Martinez D, Forns J, Guxens M, Julvez J (2010). Prenatal exposure to cell phone use and neurodevelopment at 14 months.. Epidemiology.

[29] Polk S, Sunyer J, Muñoz-Ortiz L, Barnes M, Torrent M (2004). A prospective study of Fel d1 and Der p1 exposure in infancy and childhood wheezing.. Am J Respir Crit Care Med.

[30] Ribas-Fitó N, Júlvez J, Torrent M, Grimalt JO, Sunyer J (2007). Beneficial effects of breastfeeding on cognition regardless of DDT concentrations at birth.. Am J Epidemiol.

[31] McCarthy D (1972). Manual for the McCarthy Scales of Children's Abilities..

[32] Julvez J, Ribas-Fitó N, Torrent M, Forns M, Garcia-Esteban R (2007). Maternal smoking habits and cognitive development of children at age 4 years in a population-based birth cohort.. Int J Epidemiol.

[33] Morales E, Julvez J, Torrent M, de Cid R, Guxens M (2009). Association of early-life exposure to household gas appliances and indoor nitrogen dioxide with cognition and attention behavior in preschoolers.. Am J Epidemiol.

[34] Moltó-Puigmartí C, Castellote AI, López-Sabater MC (2007). Conjugated linoleic acid determination in human milk by fast-gas chromatography.. Anal Chim Acta.

[35] Gieger C, Geistlinger L, Altmaier E, Hrabé de Angelis M, Kronenberg F (2008). Genetics meets metabolomics: a genome-wide association study of metabolite profiles in human serum.. PLoS Genet.

[36] Warensjö E, Rosell M, Hellenius ML, Vessby B, De Faire U (2009). Associations between estimated fatty acid desaturase activities in serum lipids and adipose tissue in humans: links to obesity and insulin resistance.. Lipids Health Dis.

[37] Bokor S, Dumont J, Spinneker A, Gonzalez-Gross M, Nova E (2010). Single nucleotide polymorphisms in the FADS gene cluster are associated with delta-5 and delta-6 desaturase activities estimated by serum fatty acid ratios.. J Lipid Res.

[38] Ribas-Fitó N, Cardo E, Sala M, Eulàlia de Muga M, Mazón C (2003). Breastfeeding, exposure to organochlorine compounds, and neurodevelopment in infants.. Pediatrics.

[39] González JR, Armengol L, Solé X, Guinó E, Mercader JM (2007). SNPassoc: an R package to perform whole genome association studies.. Bioinformatics.

[40] Rodriguez-Cruz M, Tovar AR, Palacios-González B, Del Prado M, Torres N (2006). Synthesis of long-chain polyunsaturated fatty acids in lactating mammary gland: role of Delta5 and Delta6 desaturases, SREBP-1, PPARalpha, and PGC-1.. J Lipid Res.

[41] Xiang M, Alfven G, Blennow M, Trygg M, Zetterstrom R (2000). Longchain polyunsaturated fatty acids in human milk and brain growth during early infancy.. Acta Paediatr.

[42] Lattka E, Eggers S, Moeller G, Heim K, Weber M (2010). A common FADS2 promoter polymorphism increases promoter activity and facilitates binding of transcription factor ELK1.. J Lipid Res.

[43] Koletzko B, Lien E, Agostoni C, Böhles H, Campoy C (2008). The roles of long-chain polyunsaturated fatty acids in pregnancy, lactation and infancy: review of current knowledge and consensus recommendations.. J Perinat Med.

[44] Caspi A, Williams B, Kim-Cohen J, Craig IW, Milne BJ (2007). Moderation of breastfeeding effects on the IQ by genetic variation in fatty acid metabolism.. Proc Natl Acad Sci U S A.

[45] Leonard AE, Kelder B, Bobik EG, Chuang LT, Lewis CJ (2002). Identification and expression of mammalian long-chain PUFA elongation enzymes.. Lipids.

[46] Kothapalli KS, Anthony JC, Pan BS, Hsieh AT, Nathanielsz PW (2007). Differential cerebral cortex transcriptomes of baboon neonates consuming moderate and high docosahexaenoic acid formulas.. PLoS One.

[47] Posthuma D, Luciano M, Geus EJ, Wright MJ, Slagboom PE (2005). A genomewide scan for intelligence identifies quantitative trait loci on 2q and 6p.. Am J Hum Genet.

[48] Thomas D (2010). Gene-environment-wide association studies: emerging approaches.. Nat Rev Genet.

[49] Sorgi PJ, Hallowell EM, Hutchins HL, Sears B (2007). Effects of an open-label pilot study with high-dose EPA/DHA concentrates on plasma phospholipids and behavior in children with attention deficit hyperactivity disorder.. Nutr J.

[50] Brookes KJ, Chen W, Xu X, Taylor E, Asherson P (2006). Association of fatty acid desaturase genes with attention-deficit/hyperactivity disorder.. Biol Psychiatry.

