# Supplementary material for: Genetic Variants of the FADS Gene Cluster and ELOVL Gene Family, Colostrums LC-PUFA Levels, Breastfeeding, and Child Cognition
Source: PLoS One. 2011 Feb 23;6(2):e17181. doi: 10.1371/journal.pone.0017181 (PMC3044172; doi:10.1371/journal.pone.0017181)
Supplement: Appendix S1 — Supporting methods. (DOC) [file pone.0017181.s001.doc]

**Genotyping procedure**

The design included tag SNPs and putative functional variants or variants previously described as being associated with LC-PUFA levels or different phenotypes, and located in the *FADS* gene cluster, *ELVOL2* and *ELOVL5* genes (1-,2,3,4). Tag SNPs were obtained from the CEU HapMap data (Rel24/phase II Nov08, NCBIB36 dbSNP126) including at least 5 kb upstream and downstream the genes and filtering for a minor allele frequency (MAF)>0.05, a Hardy-Weinberg equilibrium (HWE) p value<0.05, and a genotyping rate>75%. The tagSNP selection was performed using the pairtagging strategy (r^2^<0.8) implemented in Haploview (5). Four selftag SNPs were excluded from the design due to technical issues. Finally, thirty-six SNPs (19 in the *FADS* gene cluster, 7 in *ELOVL2* and 10 in *ELOVL5* genes) were genotyped with the SNPlex Genotyping System (Applied Biosystems) (Table S1). Positive controls (a HapMap trio) and a negative control were included in each 96-plate giving consistent results. Ten INMA-Menorca samples were duplicated and only one genotype out of 360 (0.2%) was found to be inconsistent.

In the INMA-Menorca cohort genotyping failed for five SNPs (rs412334, rs422249, rs7744440, rs209494, rs209505), and four deviated from HWE (rs968567, rs174575, rs174627 and rs13966). In the INMA-Sabadell cohort, the same 5 SNPs as in the INMA-Menorca, along with 4 more (rs2851682, rs13966, rs174455, rs472031), also failed genotyping in cord blood and maternal DNAs. One SNP (rs174575) accumulated six Mendelian errors and was deleted from the analysis. The rs761179 was in strong disequilibrium with rs2397142 (r^2^=0.99), thus it was also excluded from the analysis.

**References**

1.Tanaka T, et al. (2009) Genome-wide association study of plasma polyunsaturated fatty acids in the InCHIANTI Study. *PLoS Genet* 5:e1000338

2. Gieger C, et al. (2008) Genetics meets metabolomics: a genome-wide association study of metabolite profiles in human serum. *PLoS Genet* 4:e1000282

3. Caspi A, et al. (2007) Moderation of breastfeeding effects on the IQ by genetic variation in fatty acid metabolism. *Proc Natl Acad Sci USA* 104:18860-18865.

4. Brookes KJ, Chen W, Xu X, Taylor E, Asherson P (2006) Association of fatty acid desaturase genes with attention-deficit/hyperactivity disorder. *Biol Psychiatry* 60:1053-1061.

5. Barrett JC, Fry B, Maller J, Daly MJ (2005) Haploview: analysis and visualization of LD and haplotype maps. *Bioinformatics* 21:263-265.
