# Supplementary material for: Genetic Variants of the FADS Gene Cluster and ELOVL Gene Family, Colostrums LC-PUFA Levels, Breastfeeding, and Child Cognition
Source: PLoS One. 2011 Feb 23;6(2):e17181. doi: 10.1371/journal.pone.0017181 (PMC3044172; doi:10.1371/journal.pone.0017181)
Supplement: Figure S1 — The n-6 and n-3 fatty acid metabolism pathways. (DOC) [file pone.0017181.s002.doc]

Linoleic acid (LA)

C18:2n-6

α-Linolenic acid (ALA)

C18:3n-3

Eicosatrienoic acid (DGLA)

C20:3n-6

Arachidonic acid (AA)

C20:4n-6

ELOVL5

Docosatetraenoic acid

C22:4n-6

**ELOVL5**

**(elongation)**

Docosapentanoic acid

C22:5n-6

**FADS1**

**(delta-5 desaturation)**

20:2n-6

FADS2

(delta-8 desaturation)

**ELOVL5**

**(elongation)**

**FADS2**

**(delta-6 desaturation)**

C24:4n-6

C24:5n-6

**FADS2**

**(delta-6 desaturation)**

20:3n-3

γ-Linoleic acid (GLA)

C18:3n-6

Stearidonic acid (STD)

C18:4n-3

FADS2

(delta-8 desaturation)

Eicosatetraenoic acid (ETA)

C20:4n-3

Eicosapentanoic acid (EPA)

C20:5n-3

**ELOVL2 (elongation)**

**ELOVL2 (elongation)**

Docosapentaenoic acid (DPA)

C22:5n-3

C24:5n-3

**FADS2**

**(delta-6 desaturation)**

C24:6n-3

Docosahexaenoic acid (DHA)

C22:6n-3
