# Supplementary material for: Genetic Variants of the FADS Gene Cluster and ELOVL Gene Family, Colostrums LC-PUFA Levels, Breastfeeding, and Child Cognition
Source: PLoS One. 2011 Feb 23;6(2):e17181. doi: 10.1371/journal.pone.0017181 (PMC3044172; doi:10.1371/journal.pone.0017181)
Supplement: Table S1 — Characteristics of the SNPs analyzed in the FADS gene cluster, ELOVL2 and ELOVL5 genes. Abbreviations: SNP: single-nucleotide polymorphism; HWE: Hardy-Weinberg equilibrium; MAF: Minor allele frequency; ME: Mendelian errors. (DOC) [file pone.0017181.s003.doc]

| **SNP** | **Gene** | **Position** | **Location** | **Chr** | **Major/minor**  **allele** |  | **INMA Menorca cohort** | | |  | **INMA Sabadell cohort** | | | | | | |
| --- | --- | --- | --- | --- | --- | --- | --- | --- | --- | --- | --- | --- | --- | --- | --- | --- | --- |
|  |  |  |  |  |  |  |  |  |  |  | **Children** | | |  | **Mothers** | | |
|  |  |  |  |  |  |  | **Genotyping**  **success rate (%)** | **MAF** | **HWE**  **p value** |  | **Genotyping success rate (%)** | **MAF** | **HWE**  **p value** |  | **Genotyping success rate (%)** | **MAF** | **HWE**  **p value** |
| rs174537 | *FADS cluster* | 61552679 | intron 2 *C11orf9* | 11 | G/T |  | 99.8 | 0.301 | 0.195 |  | 97.0 | 0.292 | 0.136 |  | 94.7 | 0.287 | 0.605 |
| rs412334 | *FADS cluster* | 61560260 | 5'UTR *FEN1* | 11 | A/G |  | 0.0 | - | - |  | 0.0 | - | - |  | 0.0 | - | - |
| rs968567 | *FADS cluster* | 61595563 | near 5' *FADS2* | 11 | G/A |  | 100.0 | 0.145 | 0.014 |  | 96.2 | 0.131 | 0.495 |  | 91.7 | 0.126 | 1.000 |
| rs174570 | *FADS cluster* | 61597211 | intron 1 *FADS2* | 11 | C/T |  | 100.0 | 0.141 | 0.681 |  | 82.0 | 0.119 | 0.426 |  | 81.5 | 0.104 | 0.627 |
| rs174575 | *FADS cluster* | 61602002 | intron 1 *FADS2* | 11 | C/G |  | 98.3 | 0.263 | 0.009 |  | 79.2 | 0.296 | 0.342 (ME) |  | 85.0 | 0.278 | 0.579  (ME) |
| rs2072114 | *FADS cluster* | 61605214 | intron 1 *FADS2* | 11 | A/G |  | 99.5 | 0.104 | 0.600 |  | 97.7 | 0.108 | 0.286 |  | 94.6 | 0.116 | 0.211 |
| rs2851682 | *FADS cluster* | 61616011 | intron 5 *FADS2* | 11 | A/G |  | 99.8 | 0.072 | 0.709 |  | 0.0 | - | - |  | 0.0 | - | - |
| rs174602 | *FADS cluster* | 61624413 | intron 4 *FADS2* | 11 | A/G |  | 99.8 | 0.215 | 0.302 |  | 95.7 | 0.221 | 0.455 |  | 92.5 | 0.248 | 0.085 |
| rs526126 | *FADS cluster* | 61624884 | intron 6 *FADS2* | 11 | C/G |  | 99.0 | 0.186 | 0.321 |  | 63.5 | 0.190 | 0.837 |  | 79.4 | 0.163 | 0.087 |
| rs174626 | *FADS cluster* | 61637056 | near 3' *FADS2* | 11 | T/C |  | 99.8 | 0.475 | 0.231 |  | 96.7 | 0.488 | 0.183 |  | 94.4 | 0.492 | 0.235 |
| rs174627 | *FADS cluster* | 61637465 | near 3' *FADS2* | 11 | C/T |  | 100.0 | 0.146 | 0.004 |  | 98 | 0.136 | 0.388 |  | 94.4 | 0.122 | 1.000 |
| rs472031 | *FADS cluster* | 61638419 | near 3' *FADS3* | 11 | C/T |  | 100.0 | 0.106 | 0.431 |  | 0.0 | - | - |  | 0.0 | - | - |
| rs422249 | *FADS cluster* | 61639487 | near 3' *FADS3* | 11 | C/T |  | 0.0 | - | - |  | 0.0 | - | - |  | 0.0 | - | - |
| rs7482316 | *FADS cluster* | 61640197 | near 3' *FADS3* | 11 | A/G |  | 99.3 | 0.105 | 0.787 |  | 89.3 | 0.088 | 1.000 |  | 89.6 | 0.087 | 0.786 |
| rs174455 | *FADS cluster* | 61656116 | intron 1 *FADS3* | 11 | A/G |  | 99.5 | 0.343 | 0.580 |  | 0.0 | - | - |  | 0.0 | - | - |
| rs174464 | *FADS cluster* | 61657925 | intron 1 *FADS3* | 11 | C/T |  | 99.3 | 0.243 | 0.892 |  | 81.2 | 0.289 | 0.101 |  | 82.5 | 0.293 | 0.272 |
| rs528285 | *FADS cluster* | 61660703 | near 5' *FADS3* | 11 | C/T |  | 99.0 | 0.324 | 0.568 |  | 11.7 | 0.326 | 1.000 |  | 2.4 | 0.429 | 0.620 |
| rs174468 | *FADS cluster* | 61663690 | near 5' *FADS3* | 11 | G/A |  | 99.8 | 0.452 | 0.614 |  | 97.2 | 0.416 | 1.000 |  | 93.4 | 0.401 | 0.375 |
| rs13966 | *FADS cluster* | 61664991 | 3' *RAB3IL1* | 11 | C/T |  | 98.0 | 0.455 | 0.008 |  | 0.0 | - | - |  | 0.0 | - | - |
| rs3734397 | *ELOVL2* | 10982847 | 3'UTR | 6 | A/G |  | 99.3 | 0.296 | 0.904 |  | 97.7 | 0.252 | 0.344 |  | 94.9 | 0.268 | 0.160 |
| rs953413 | *ELOVL2* | 11012858 | intron 1 | 6 | G/A |  | 98.5 | 0.426 | 0.682 |  | 84.0 | 0.412 | 0.140 |  | 90.3 | 0.465 | 0.662 |
| rs10498676 | *ELOVL2* | 11026998 | intron 1 | 6 | G/A |  | 100.0 | 0.168 | 0.478 |  | 88.3 | 0.129 | 0.336 |  | 93.0 | 0.154 | 0.512 |
| rs6936315 | *ELOVL2* | 11035971 | intron 1 | 6 | T/C |  | 97.5 | 0.155 | 1.000 |  | 72.8 | 0.143 | 0.144 |  | 79.9 | 0.145 | 1.000 |
| rs3798719 | *ELOVL2* | 11036824 | intron 1 | 6 | C/T |  | 100.0 | 0.277 | 0.620 |  | 92.1 | 0.281 | 0.068 |  | 90.8 | 0.295 | 0.466 |
| rs7744440 | *ELOVL2* | 11038510 | intron 1 | 6 | G/T |  | 0.0 | - | - |  | 0.0 | - | - |  | 0.0 | - | - |
| rs13204015 | *ELOVL2* | 11040459 | intron 1 | 6 | T/C |  | 99.8 | 0.030 | 0.668 |  | 82.7 | 0.043 | 0.418 |  | 92.0 | 0.042 | 0.614 |
| rs17544159 | *ELOVL5* | 53130519 | near 3' | 6 | A/C |  | 99.8 | 0.062 | 0.184 |  | 87.1 | 0.057 | 1.000 |  | 89.8 | 0.072 | 1.000 |
| rs2281274 | *ELOVL5* | 53143553 | intron 3 | 6 | T/C |  | 98.8 | 0.224 | 0.112 |  | 95.4 | 0.278 | 0.246 |  | 93.2 | 0.280 | 0.138 |
| rs2294859 | *ELOVL5* | 53159575 | intron 2 | 6 | T/C |  | 99.3 | 0.092 | 0.230 |  | 90.1 | 0.073 | 0.708 |  | 92.2 | 0.083 | 0.402 |
| rs761179 | *ELOVL5* | 53166835 | intron 1 | 6 | T/C |  | 99.0 | 0.342 | 1.000 |  | 98.5 | 0.332 | 0.731 |  | 94.9 | 0.340 | 1.000 |
| rs9395855 | *ELOVL5* | 53176764 | intron 1 | 6 | T/G |  | 99.3 | 0.498 | 0.764 |  | 98.2 | 0.487 | 0.919 |  | 94.0 | 0.476 | 0.932 |
| rs209494 | *ELOVL5* | 53183793 | intron 1 | 6 | C/T |  | 0.0 | - | - |  | 0.0 | - | - |  | 0.0 | - | - |
| rs11968589 | *ELOVL5* | 53190233 | intron 1 | 6 | C/T |  | 99.5 | 0.088 | 0.755 |  | 91.1 | 0.135 | 0.497 |  | 92.3 | 0.115 | 0.135 |
| rs209505 | *ELOVL5* | 53192021 | intron 1 | 6 | A/G |  | 0.0 | - | - |  | 0.0 | - | - |  | 0.0 | - | - |
| rs2397142 | *ELOVL5* | 53200298 | intron 1 | 6 | C/G |  | 98.8 | 0.342 | 0.911 |  | 88.6 | 0.322 | 0.625 |  | 93.9 | 0.341 | 0.924 |
| rs12207094 | *ELOVL5* | 53204174 | intron 1 | 6 | A/T |  | 99.8 | 0.141 | 0.678 |  | 96.2 | 0.131 | 0.652 |  | 94.0 | 0.143 | 0.861 |
