# Supplementary material for: Genetic Variants of the FADS Gene Cluster and ELOVL Gene Family, Colostrums LC-PUFA Levels, Breastfeeding, and Child Cognition
Source: PLoS One. 2011 Feb 23;6(2):e17181. doi: 10.1371/journal.pone.0017181 (PMC3044172; doi:10.1371/journal.pone.0017181)
Supplement: Table S2 — Associations* between maternal genetic variants in the FADS cluster, ELOLV2 and ELOVL5 genes and levels of LC-PUFA in colostrum. *p-value of the association using additive genetic models. Change in concentrations in the additive genetic models assuming a trend per copy of the minor allele. MAF: Minor allele frequency; AA: Arachidonic acid; EPA: Eicosapentanoic acid; DHA: Docosahexaenoic acid. (DOC) [file pone.0017181.s004.doc]

|  | **Major/minor**  **allele** | **MAF** |  | **AA** | **P value** |  | **EPA** | **P value** |  | **DHA** | **P value** |  | **EPA/AA**  **ratio** | **P value** |  | **DHA/AA**  **ratio** | **P value** |
| --- | --- | --- | --- | --- | --- | --- | --- | --- | --- | --- | --- | --- | --- | --- | --- | --- | --- |
|  |  |  |  |  |  |  |  |  |  |  |  |  |  |  |  |  |  |
| ***FADS* cluster** |  |  |  |  |  |  |  |  |  |  |  |  |  |  |  |  |  |
| rs174537 | G/T | 0.287 |  | -10% | 2e-04 |  |  | 0.329 |  | -7% | 0.057 |  |  | 0.150 |  |  | 0.136 |
| rs968567 | G/A | 0.126 |  |  | 0.088 |  |  | 0.562 |  |  | 0.494 |  |  | 0.105 |  |  | 0.504 |
| rs174570 | C/T | 0.104 |  | -13% | 0.0003 |  |  | 0.290 |  |  | 0.197 |  |  | 0.204 |  | -9% | 0.051 |
| rs2072114 | A/G | 0.116 |  | -10% | 0.006 |  |  | 0.078 |  | -11% | 0.055 |  |  | 0.799 |  |  | 0.938 |
| rs174602 | A/G | 0.248 |  | -6% | 0.012 |  | -7% | 0.022 |  | -12% | 0.0006 |  |  | 0.482 |  | -6% | 0.052 |
| rs526126 | C/G | 0.163 |  | -9% | 0.017 |  |  | 0.996 |  |  | 0.736 |  |  | 0.211 |  |  | 0.080 |
| rs174626 | T/C | 0.492 |  | -7% | 0.001 |  |  | 0.167 |  |  | 0.160 |  |  | 0.276 |  |  | 0.152 |
| rs174627 | C/T | 0.122 |  |  | 0.066 |  |  | 0.118 |  | -9% | 0.066 |  |  | 0.959 |  |  | 0.724 |
| rs7482316 | A/G | 0.087 |  |  | 0.136 |  |  | 0.147 |  |  | 0.261 |  | +16% | 0.008 |  | +15% | 0.001 |
| rs174464 | C/T | 0.293 |  | -7% | 0.004 |  |  | 0.085 |  | -9% | 0.015 |  |  | 0.674 |  |  | 0.720 |
| rs174468 | G/A | 0.401 |  | +6% | 0.015 |  |  | 0.380 |  |  | 0.920 |  |  | 0.330 |  | -6% | 0.019 |
|  |  |  |  |  |  |  |  |  |  |  |  |  |  |  |  |  |  |
| ***ELOVL2*** |  |  |  |  |  |  |  |  |  |  |  |  |  |  |  |  |  |
| rs3734397 | A/G | 0.268 |  |  | 0.144 |  |  | 0.060 |  |  | 0.532 |  | -11% | 0.005 |  |  | 0.583 |
| rs953413 | G/A | 0.465 |  |  | 0.394 |  | +10% | 0.011 |  |  | 0.524 |  | +12% | 0.004 |  |  | 0.124 |
| rs10498676 | G/A | 0.154 |  |  | 0.090 |  |  | 0.822 |  |  | 0.230 |  |  | 0.393 |  |  | 0.910 |
| rs6936315 | T/C | 0.145 |  |  | 0.851 |  |  | 0.366 |  |  | 0.255 |  |  | 0.267 |  |  | 0.183 |
| rs3798719 | C/T | 0.295 |  |  | 0.715 |  | +10% | 0.011 |  |  | 0.179 |  | +8% | 0.037 |  |  | 0.206 |
| rs13204015 | T/C | 0.042 |  |  | 0.757 |  |  | 0.793 |  |  | 0.732 |  |  | 0.884 |  |  | 0.556 |
|  |  |  |  |  |  |  |  |  |  |  |  |  |  |  |  |  |  |
| ***ELOVL5*** |  |  |  |  |  |  |  |  |  |  |  |  |  |  |  |  |  |
| rs17544159 | A/C | 0.072 |  |  | 0.317 |  |  | 0.345 |  |  | 0.757 |  | +18% | 0.022 |  |  | 0.072 |
| rs2281274 | T/C | 0.280 |  |  | 0.631 |  |  | 0.613 |  |  | 0.293 |  |  | 0.746 |  |  | 0.317 |
| rs2294859 | T/C | 0.083 |  |  | 0.781 |  |  | 0.228 |  |  | 0.239 |  |  | 0.121 |  |  | 0.074 |
| rs9395855 | T/G | 0.476 |  |  | 0.644 |  |  | 0.219 |  |  | 0.811 |  | +8% | 0.040 |  |  | 0.283 |
| rs11968589 | C/T | 0.115 |  |  | 0.240 |  |  | 0.924 |  |  | 0.274 |  |  | 0.538 |  |  | 0.690 |
| rs2397142 | C/G | 0.341 |  |  | 0.661 |  |  | 0.314 |  |  | 0.947 |  |  | 0.091 |  |  | 0.419 |
| rs12207094 | A/T | 0.143 |  |  | 0.313 |  |  | 0.060 |  |  | 0.380 |  | +16% | 0.002 |  | +9 | 0.027 |
