# Supplementary material for: Genetic Variants of the FADS Gene Cluster and ELOVL Gene Family, Colostrums LC-PUFA Levels, Breastfeeding, and Child Cognition
Source: PLoS One. 2011 Feb 23;6(2):e17181. doi: 10.1371/journal.pone.0017181 (PMC3044172; doi:10.1371/journal.pone.0017181)
Supplement: Table S3 — Associations* between child polymorphisms in the FADS cluster, ELOVL2 and ELOVL5 genes and child cognition by cohort. *p values in the additive genetic models assuming a trend per copy of the minor allele. MAF: Minor allele frequency. (DOC) [file pone.0017181.s005.doc]

|  | **INMA Menorca cohort** | |  | **INMA Sabadell cohort** | |
| --- | --- | --- | --- | --- | --- |
|  | MAF | p value |  | MAF | p value |
| ***FADS* cluster** |  |  |  |  |  |
| rs174537 | 0.301 | 0.929 |  | 0.292 | 0.298 |
| rs968567 | 0.145 | - |  | 0.131 | 0.610 |
| rs174570 | 0.141 | 0.698 |  | 0.119 | 0.399 |
| rs2072114 | 0.104 | 0.800 |  | 0.108 | 0.977 |
| rs2851682 | 0.072 | 0.490 |  | - | - |
| rs174602 | 0.215 | 0.605 |  | 0.221 | 0.740 |
| rs526126 | 0.186 | 0.379 |  | 0.190 | 0.483 |
| rs174626 | 0.475 | 0.190 |  | 0.488 | 0.773 |
| rs472031 | 0.106 | 0.590 |  | - | - |
| rs174627 | - | - |  | 0.122 | 0.890 |
| rs7482316 | 0.105 | 0.048 |  | 0.088 | 0.109 |
| rs174464 | 0.243 | 0.565 |  | 0.289 | 0.394 |
| rs174468 | 0.452 | 0.461 |  | 0.416 | 0.548 |
|  |  |  |  |  |  |
| ***ELOVL2*** |  |  |  |  |  |
| rs3734397 | 0.296 | 0.297 |  | 0.252 | 0.500 |
| rs953413 | 0.426 | 0.263 |  | 0.412 | 0.791 |
| rs10498676 | 0.168 | 0.300 |  | 0.129 | 0.940 |
| rs6936315 | 0.155 | 0.593 |  | 0.143 | 0.346 |
| rs3798719 | 0.277 | 0.132 |  | 0.281 | 0.426 |
| rs13204015 | 0.030 | 0.457 |  | - | - |
|  |  |  |  |  |  |
| ***ELOVL5*** |  |  |  |  |  |
| rs17544159 | 0.062 | 0.331 |  | 0.057 | 0.315 |
| rs2281274 | 0.224 | 0.116 |  | 0.278 | 0.296 |
| rs2294859 | 0.092 | 0.111 |  | 0.073 | - |
| rs9395855 | 0.498 | 0.060 |  | 0.487 | 0.793 |
| rs11968589 | 0.088 | 0.037 |  | 0.135 | 0.242 |
| rs2397142 | 0.342 | 0.377 |  | 0.322 | 0.999 |
| rs12207094 | 0.141 | 0.850 |  | 0.131 | 0.274 |
