# Supplementary material for: Genetic Variants of the FADS Gene Cluster and ELOVL Gene Family, Colostrums LC-PUFA Levels, Breastfeeding, and Child Cognition
Source: PLoS One. 2011 Feb 23;6(2):e17181. doi: 10.1371/journal.pone.0017181 (PMC3044172; doi:10.1371/journal.pone.0017181)
Supplement: Table S4 — Association of child genetic polymorphisms in the FADS cluster (rs174468) and ELOVL5 (rs2397142) with general cognitive function score and selected covariates by cohort. ¶ Unless otherwise specified, p value derived from chi-2 test; §p value derived from Kruskall-Wallis test. (DOC) [file pone.0017181.s006.doc]

|  | **rs174468** | |  |  | **rs2397142** | |  |
| --- | --- | --- | --- | --- | --- | --- | --- |
| **INMA Menorca cohort** | **GG** | **AG-AA** | **p value**^¶^ |  | **CC** | **CG-GG** | **p value**^¶^ |
|  | **N=108** | **N=232** |  |  | **N=160** | **N=207** |  |
| **General cognitive (mean, sd)** | 99.7 (14.6) | 99.4 (15.1) | 0.867^§^ |  | 100.3 (15.2) | 98.7 (14.5) | 0.283^§^ |
| **Breastfeeding, yes (%)** | 82.4 | 83.2 | 0.858 |  | 85.9 | 81.6 | 0.259 |
| **Birth weight (mean, sd)** | 3156.6 (479.7) | 3234.0 (490.9) | 0.174^§^ |  | 3210.3 | 3200.4 | 0.845^§^ |
| **Gestational age, weeks (mean, sd)** | 39.4 (1.9) | 39.2 (1.6) | 0.360^§^ |  | 39.2 (1.7) | 39.3 (1.7) | 0.632^§^ |
| **Preterm (<37 weeks) (%)** | 3.7 | 6.0 | 0.372 |  | 5.5 | 5.2 | 0.904 |
| **Maternal social class (%)** |  |  |  |  |  |  |  |
| Professional | 21.5 | 14.4 | 0.050 |  | 15.7 | 17.5 | 0.468 |
| Skilled manual & non-manual | 71.9 | 71.2 |  |  | 69.9 | 72.5 |  |
| Partially skilled & unskilled | 6.5 | 14.4 |  |  | 14.4 | 10.0 |  |
| **Maternal education (%)** |  |  |  |  |  |  |  |
| High | 20.4 | 12.9 | 0.207 |  | 15.8 | 15.0 |  |
| Secondary | 25.9 | 28.0 |  |  | 28.8 | 25.9 |  |
| Primary or less | 53.7 | 59.1 |  |  | 55.5 | 59.1 |  |
| **Maternal smoking in pregnancy, yes (%)** | 22.2 | 20.3 | 0.678 |  | 20.0 | 21.7 | 0.685 |
| **Maternal alcohol in pregnancy, yes (%)** | 20.4 | 21.6 | 0.804 |  | 16.9 | 24.2 | 0.089 |
| **Gas cooker, yes (%)** | 77.8 | 71.1 | 0.197 |  | 74.7 | 72.0 | 0.587 |
|  |  |  |  |  |  |  |  |
|  | **rs174468** | |  |  | **rs2397142** | |  |
| **INMA Sabadell cohort** | **GG** | **AG-AA** | **p value**^¶^ |  | **CC** | **CG-GG** | **p value**^¶^ |
|  | **N=103** | **N=170** |  |  | **N=119** | **N=144** |  |
| **General cognitive (mean, sd)** | 98.4 (15.7) | 101.2 (15.2) | 0.154^§^ |  | 100.5 (15.6) | 99.4 (15.4) | 0.565^§^ |
| **Breastfeeding, yes (%)** | 82.5 | 80.0 | 0.607 |  | 79.0 | 82.6 | 0.453 |
| **Birth weight (mean, sd)** | 3247.9 (433.5) | 3240.0 (29.6) | 0.875^§^ |  | 3220.7 (391.7) | 3262.8 (412.9) | 0.400^§^ |
| **Gestational age, weeks (mean, sd)** | 39.9 (1.5) | 39.8 (1.3) | 0.598^§^ |  | 40.0 (1.2) | 39.8 (1.4) | 0.114^§^ |
| **Preterm (<37 weeks) (%)** | 4.8 | 1.2 | 0.065 |  | 0.8 | 3.5 | 0.155 |
| **Maternal social class (%)** |  |  |  |  |  |  |  |
| Professional | 12.8 | 6.7 | 0.206 |  | 13.8 | 5.0 | 0.107 |
| Skilled manual & non-manual | 25.6 | 21.0 |  |  | 20.7 | 25.0 |  |
| Partially skilled & unskilled | 61.5 | 72.3 |  |  | 65.5 | 70.0 |  |
| **Maternal education (%)** |  |  |  |  |  |  |  |
| High | 33.0 | 27.1 | 0.203 |  | 26.9 | 30.6 | 0.615 |
| Secondary | 47.6 | 44.1 |  |  | 43.7 | 45.1 |  |
| Primary or less | 19.4 | 28.8 |  |  | 29.4 | 24.3 |  |
| **Maternal smoking in pregnancy, yes (%)** | 15.5 | 14.1 | 0.748 |  | 18.5 | 11.2 | 0.095 |
| **Maternal alcohol in pregnancy, yes (%)** | 9.7 | 12.3 | 0.504 |  | 12.6 | 10.5 | 0.592 |
| **Gas cooker, yes (%)** | 53.4 | 60.6 | 0.244 |  | 62.2 | 56.3 | 0.330 |
