# Supplementary material for: Genetic Variants of the FADS Gene Cluster and ELOVL Gene Family, Colostrums LC-PUFA Levels, Breastfeeding, and Child Cognition
Source: PLoS One. 2011 Feb 23;6(2):e17181. doi: 10.1371/journal.pone.0017181 (PMC3044172; doi:10.1371/journal.pone.0017181)
Supplement: Table S5 — Comparison of the distribution of child and maternal characteristics between children included and not included in the study by cohort. ¶ Unless otherwise specified, p value derived from chi-2 test; §p value derived from Kruskall-Wallis test. (DOC) [file pone.0017181.s007.doc]

|  | **INMA Menorca cohort** | | |  | **INMA Sabadell cohort** | | |
| --- | --- | --- | --- | --- | --- | --- | --- |
|  | **Included** | **Not Included** | **p value**^¶^ |  | **Included** | **Not included** | **p value**^¶^ |
| **Child gender, female (%)** | 51.5 | 40.0 | 0.114 |  | 50.2 | 52.1 | 0.661 |
| **Birth weight (g), mean (sd)** | 3209.4 (488.0) | 3131.6 (461.3) | 0.270^§^ |  | 3241.9 (403.7) | 3238.3 (457.2) | 0.902^§^ |
| **Preterm (<37 weeks) (%)** | 5.3 | 7.3 | 0.553 |  | 2.6 | 2.7 | 0.905 |
| **General cognition, mean (sd)** | 99.5 (15.0) | 98.3 (13.2) | 0.685^§^ |  | 100.1 (15.4) | 100.5 (15.1) | 0.810^§^ |
| **Breastfeeding (%)** | 82.9 | 85.5 | 0.643 |  | 80.9 | 74.5 | 0.078 |
| **Maternal age (years), mean (sd)** | 29.1 (4.3) | 28.2 (4.8) | 0.177^§^ |  | 31.5 (4.1) | 31.7 (4.2) | 0.602^§^ |
| **Maternal education, (%)** |  |  |  |  |  |  |  |
| Primary or less | 57.4 | 65.0 | 0.086 |  | 25.3 | 32.6 | 0.116 |
| Secondary | 27.4 | 32.5 |  |  | 45.4 | 37.9 |  |
| University | 15.3 | 2.5 |  |  | 29.3 | 29.6 |  |
| **Maternal social class, (%)** |  |  |  |  |  |  |  |
| Professional | 16.7 | 11.5 | 0.410 |  | 14.5 | 9.1 | 0.228 |
| Skilled manual & non-manual | 71.4 | 71.1 |  |  | 19.8 | 22.8 |  |
| Partially skilled & unskilled | 11.9 | 17.3 |  |  | 65.7 | 68.0 |  |
| **Maternal smoking in pregnancy, yes (%)** | 20.9 | 21.8 | 0.874 |  | 14.7 | 17.1 | 0.481 |
| **Maternal alcohol in pregnancy, yes (%)** | 21.2 | 20.4 | 0.893 |  | 11.4 | 14.8 | 0.214 |
| **Gas cooker, yes (%)** | 73.2 | 78.2 | 0.438 |  | 57.5 | 60.6 | 0.499 |
