# Supplementary material for: Genetic Variants of the FADS Gene Cluster and ELOVL Gene Family, Colostrums LC-PUFA Levels, Breastfeeding, and Child Cognition
Source: PLoS One. 2011 Feb 23;6(2):e17181. doi: 10.1371/journal.pone.0017181 (PMC3044172; doi:10.1371/journal.pone.0017181)
Supplement: Supplementary file 8 [file pone.0017181.s008.doc]

	rs174537	rs174570	rs2072114	rs2851682	rs174602	rs526126	rs174626	rs472031	rs7482316	rs174455	rs174464	rs528285
rs174537												
rs174570	1/0.35											
rs2072114	0.932/0.23	0.52/0.194										
rs2851682	0.929/0.149	0.887/0.367	0.937/0.567									
rs174602	0.496/0.156	0.409/0.099	0.199/0.017	1/0.021								
rs526126	0.61/0.193	0.122/0.011	0.483/0.006	0.689/0.008	0.317/0.083							
rs174626	0.763/0.279	0.63/0.071	0.602/0.046	0.365/0.011	0.698/0.147	0.84/0.175						
rs472031	0.674/0.024	0.48/0.005	1/0.014	1/0.009	0.695/0.016	0.733/0.015	1/0.134					
rs7482316	0.699/0.125	0.688/0.327	0.288/0.079	0.49/0.162	0.427/0.075	0.417/0.086	1/0.124	1/0.014				
rs174455	0.622/0.321	0.568/0.100	0.534/0.063	0.323/0.015	0.622/0.203	0.697/0.210	0.935/0.503	0.855/0.047	0.9/0.175			
rs174464	0.484/0.174	0.018/0.000	0.249/0.022	0.54/0.007	0.329/0.092	0.344/0.082	0.948/0.321	0.875/0.003	1/0.037	1/0.620		
rs528285	0.034/0.001	0.141/0.007	0.729/0.129	0.778/0.096	0.045/0.001	0.639/0.045	0.564/0.168	0.932/0.215	0.236/0.013	0.066/0.004	0.041/0.001	
rs174468	0.688/0.167	0.72/0.068	0.641/0.039	0.568/0.020	0.767/0.131	0.713/0.093	0.889/0.582	0.956/0.090	1/0.092	0.969/0.398	1/0.263	1/0.391
