# Supplementary material for: Genetic Variants of the FADS Gene Cluster and ELOVL Gene Family, Colostrums LC-PUFA Levels, Breastfeeding, and Child Cognition
Source: PLoS One. 2011 Feb 23;6(2):e17181. doi: 10.1371/journal.pone.0017181 (PMC3044172; doi:10.1371/journal.pone.0017181)
Supplement: Supplementary file 9 [file pone.0017181.s009.doc]

	rs3734397	rs953413	rs10498676	rs6936315	rs3798719
rs3734397					
rs953413	1/0.317				
rs10498676	1/0.087	1/0.269			
rs6936315	0.623/0.030	0.718/0.073	0.886/0.030		
rs3798719	1/0.161	0.981/0.492	0.401/0.013	0.723/0.037	
rs13204015	1/0.013	1/0.024	1/0.0060	1/0.169	1/0.012
