# Supplementary material for: Genetic Variants of the FADS Gene Cluster and ELOVL Gene Family, Colostrums LC-PUFA Levels, Breastfeeding, and Child Cognition
Source: PLoS One. 2011 Feb 23;6(2):e17181. doi: 10.1371/journal.pone.0017181 (PMC3044172; doi:10.1371/journal.pone.0017181)
Supplement: Supplementary file 10 [file pone.0017181.s010.doc]

	rs17544159	rs2281274	rs2294859	rs761179	rs9395855	rs11968589	rs2397142
rs17544159							
rs2281274	0.444/0.046						
rs2294859	1/0.007	0.687/0.169					
rs761179	1/0.035	0.751/0.086	1/0.054				
rs9395855	1/0.069	0.344/0.035	1/0.102	0.991/0.502			
rs11968589	1/0.007	0.8/0.213	0.397/0.002	1/0.050	0.572/0.031		
rs2397142	1/0.035	0.747/0.084	1/0.054	1/0.994	1/0.510	1/0.050	
rs12207094	0.949/0.368	0.028/0.000	1/0.017	1/0.086	1/0.169	1/0.016	1/0.086
